# Suppressive Control of Incentive Salience in Real-World Human Vision

**DOI:** 10.1523/JNEUROSCI.0766-23.2023

**Published:** 2023-09-13

**Authors:** Clayton Hickey, David Acunzo, Jaclyn Dell

**Affiliations:** Centre for Human Brain Health and School of Psychology, University of Birmingham, Birmingham B15 2TT, United Kingdom

**Keywords:** attention, EEG, incentive salience, machine learning, reward

## Abstract

Reward-related activity in the dopaminergic midbrain is thought to guide animal behavior, in part by boosting the perceptual and attentional processing of reward-predictive environmental stimuli. In line with this incentive salience hypothesis, studies of human visual search have shown that simple synthetic stimuli, such as lines, shapes, or Gabor patches, capture attention to their location when they are characterized by reward-associated visual features, such as color. In the real world, however, we commonly search for members of a category of visually heterogeneous objects, such as people, cars, or trees, where category examples do not share low-level features. Is attention captured to examples of a reward-associated real-world object category? Here, we have human participants search for targets in photographs of city and landscapes that contain task-irrelevant examples of a reward-associated category. We use the temporal precision of EEG machine learning and ERPs to show that these distractors acquire incentive salience and draw attention, but do not capture it. Instead, we find evidence of rapid, stimulus-triggered attentional suppression, such that the neural encoding of these objects is degraded relative to neutral objects. Humans appear able to suppress the incentive salience of reward-associated objects when they know these objects will be irrelevant, supporting the rapid deployment of attention to other objects that might be more useful. Incentive salience is thought to underlie key behaviors in eating disorders and addiction, among other conditions, and the kind of suppression identified here likely plays a role in mediating the attentional biases that emerge in these circumstances.

**Significance Statement** Like other animals, humans are prone to notice and interact with environmental objects that have proven rewarding in earlier experience. However, it is common that such objects have no immediate strategic use and are therefore distracting. Do these reward-associated real-world objects capture our attention, despite our strategic efforts otherwise? Or are we able to strategically control the impulse to notice them? Here we use machine learning classification of human electrical brain activity to show that we can establish strategic control over the salience of naturalistic reward-associated objects. These objects draw our attention, but do not necessarily capture it, and this kind of control may play an important role in mediating conditions like eating disorder and addiction.

## Introduction

Humans and other animals preferentially approach stimuli that have been associated with positive outcome in prior experience, and this is thought to involve an impact of reward on perception and attention. By this incentive salience hypothesis, reward-elicited activity in the dopaminergic midbrain impacts perceptual systems, causing reward-predictive stimuli to become salient and attention-drawing and ensuring the information carried by these objects gains access to decision-making and motor control (Berridge and Robinson, 1998). This bias is thought to be independent of strategy, with reward-associated stimuli drawing attention even when this is inconsistent with goals.

In line with this, visual search experiments in humans have shown that irrelevant reward-associated stimuli interfere with task-focused behavior (e.g., Della Libera and Chelazzi, 2009; Hickey et al., 2009; Anderson et al., 2011; Le Pelley et al., 2015), and this has been linked to activity in dopaminergic brain nuclei (Hickey and Peelen, 2015, 2017; Barbaro et al., 2017) and to the concentration of intrasynaptic dopamine in these areas (Anderson, 2016). The representative behavioral finding is that responses to a target are slower and less accurate when the environment contains a reward-associated distractor. Although this behavioral effect is ambiguous — it is potentially a product of filtering costs and the need for cognitive control rather than the capture of attention — results from EEG and MEG have convincingly demonstrated that attention is deployed to the reward-associated stimulus by showing that reward-associated distractors elicit an N2pc (Luck and Hillyard, 1994), a component of the ERP linked to attentional selection and resolution (e.g., Hickey et al., 2009; Qi et al., 2013; Donohue et al., 2016). Similarly, MRI results have demonstrated sensitivity to reward-associated distractors in early visual cortex (Itthipuripat et al., 2019).

Importantly, this existing body of work has relied on visual search arrays composed of synthetic objects: circles, squares, lines, or Gabor patches presented in regular arrays and characterized by saturated primary colors. In roughly the same timeframe as these studies, a separate literature has demonstrated that the exclusive use of such stimuli can lead to misunderstanding of the mechanisms that support visual search (for review, see Peelen and Kastner, 2014). Naturalistic search through real-world images is faster than work with synthetic stimuli has suggested should be the case (Thorpe et al., 1996), possibly because of the constraining influence of scene semantics and gist (Torralba et al., 2006; Wolfe et al., 2011), and real world search is sensitive to issues like target and distractor familiarity (Mruczek and Sheinberg, 2005; Hershler and Hochstein, 2009) and the characteristic positioning of objects in a scene (Kaiser et al., 2019).

This motivates the need for dedicated investigation of naturalistic incentive salience. Results from experiments with scene stimuli demonstrate that examples of reward-associated real-world object categories disrupt behavioral responses to targets (Hickey et al., 2015). Multivoxel classification analysis of fMRI has shown that ventral visual cortex carries more information about a naturalistic reward-associated target than it does a neutral target, but less information about a reward-associated distractor, and this has been interpreted as evidence of the misallocation of attention (Hickey and Peelen, 2015, 2017; Barbaro et al., 2017). The idea here is that the fleeting capture of attention to the distractor is not reflected in the hemodynamic fMRI signal because of the low temporal accuracy of this measure. Instead, fMRI indexes the long-lived postcapture suppression of the distractor that allows attention to be redeployed in search for the target.

These behavioral and imaging results have therefore been interpreted as evidence that reward-associated naturalistic distractors capture attention, but this clearly rests on a pair of questionable assumptions. The first is that the behavioral cost of a reward-associated distractor in naturalistic search necessarily reflects its ability to capture attention; as noted above, the alternative is that these objects are not selected, but nevertheless degrade behavior, for example, by creating the need for attentional filtering or cognitive control (Folk and Remington, 1998; Sawaki and Luck, 2010; Gaspelin and Luck, 2019). The second assumption is that the suppression of reward-associated distractors observed in fMRI is a reaction to preceding attentional selection; the alternative is that the reward-associated distractor is suppressed from its first appearance. In the current study, we leverage the temporal precision of EEG machine-learning classification and ERPs to directly test the idea that attention is captured to examples of reward-associated distractor categories presented in photographs of real-world scenes.

## Materials and Methods

**Figure 1.**
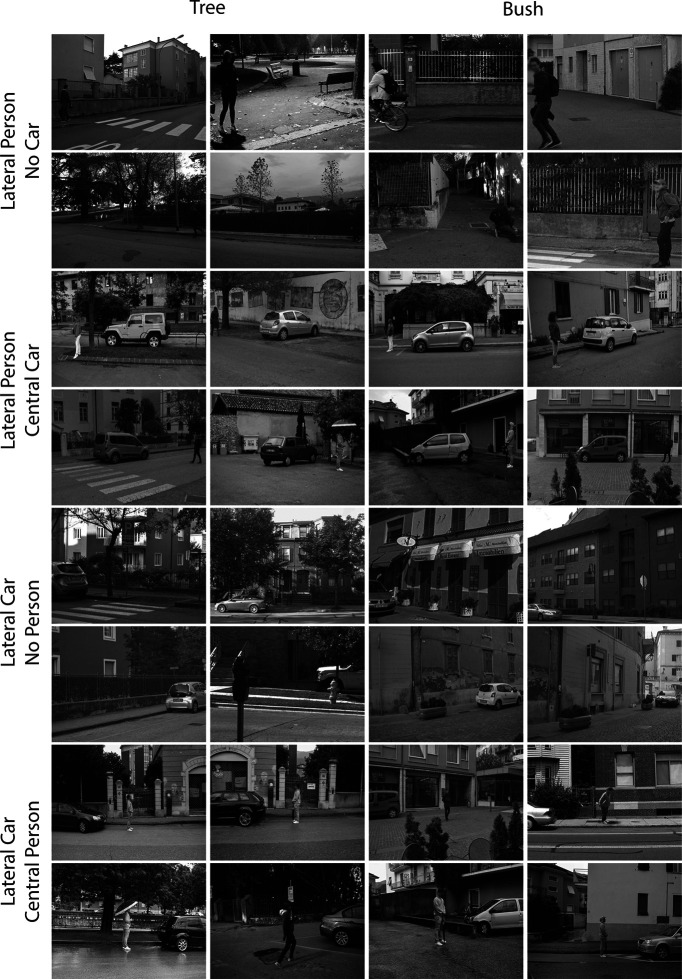
Examples of scene stimuli. The two left columns contain examples of trees; the two right columns contain examples of bushes. The rows are organized according to the presence and position of people and cars.

We had participants search through photographs of scenes for examples of real-world categories (i.e., cars, people, and plants) and report a characteristic of the target category (Fig. 1). When the target category was cars or people, participants reported the facing direction of the target; when the target was plants, they reported whether the scene contained trees or bushes.

**Figure 2.**
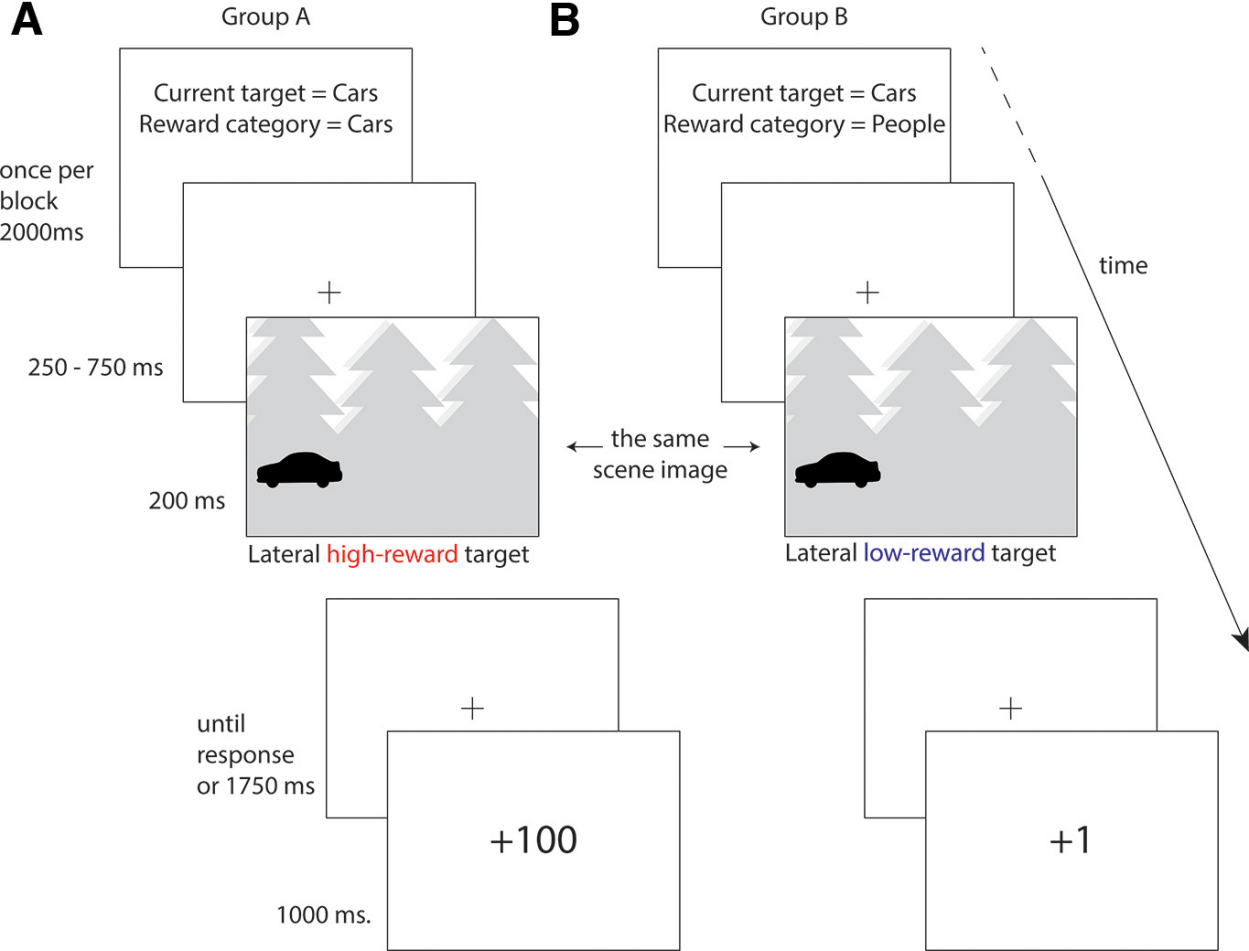
Trial examples when search is for cars. ***A***, In subject Group A, the high-reward category is cars; and in this example, the current target of search is cars. The scene contains an example of the target category; for this subject group, this is a lateral high-reward target. The task is to report the facing direction of the target, which is left, and correct response garners high-magnitude reward. ***B***, In subject Group B, the high-reward category is people; but in this example, the current target of search is cars. The scene contains an example of the target category; for this subject group, this is a lateral low-reward target. The task is to report the facing direction of the target, which is left, and correct response garners low-magnitude reward.

**Figure 3.**
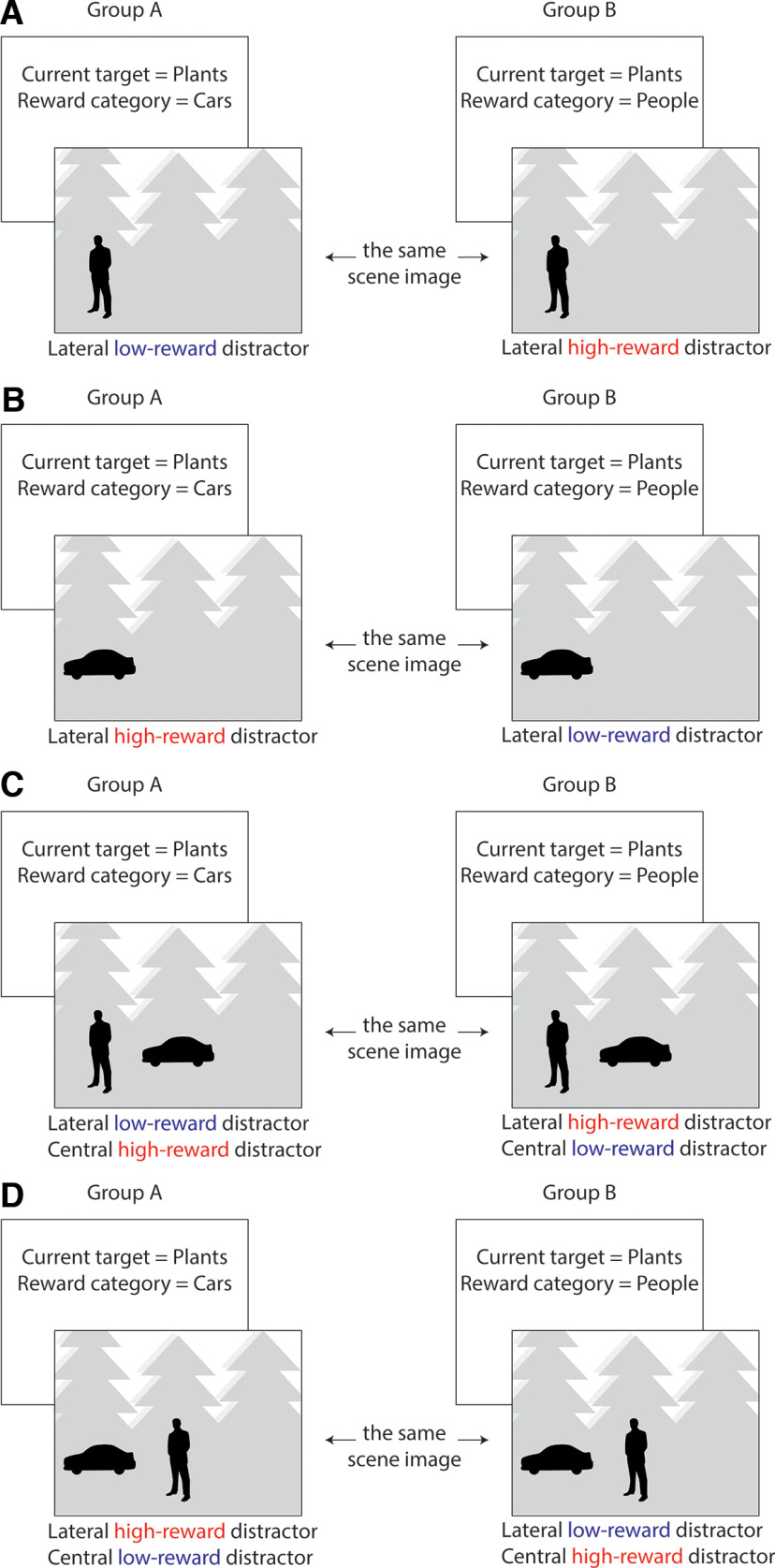
Trial examples for the critical conditions when search is for the low-reward category plants. In Group A, the high-reward category is cars; whereas in Group B, the high-reward category is people. The scenes can contain examples of people or cars, or examples of both categories concurrently. When people and cars are both present in the scene, one example is presented laterally and the other centrally. ***A***, The scene contains an example of the nontarget category people. For Group A, this scene contains a lateral low-reward distractor; whereas for Group B, this same scene contains a lateral high-reward distractor. ***B***, The scene contains an example of the nontarget category cars. For Group A, this scene contains a lateral high-reward distractor; whereas for Group B, this same scene contains a lateral low-reward distractor. ***C***, The scene contains a lateral example of the nontarget category people and a central example of the nontarget category cars. For Group A, this scene contains a lateral low-reward distractor and a central high-reward distractor. For Group B, this same scene contains a lateral high-reward distractor and a central low-reward distractor. ***D***, The scene contains a lateral example of the nontarget category cars and a central example of the nontarget category people. For Group A, this scene contains a lateral high-reward distractor and a central low-reward distractor. For Group B, this scene contains a lateral low-reward distractor and a central high-reward distractor.

For each participant, a single category — always either cars or people — was associated with reward. In blocks where this category was the cued target, correct performance earned 100 points with cash value (Fig. 2*A*). When any other category was the cued target, correct performance earned only 1 point (Fig. 2*B*). Critically, when participants were cued to search for a low-reward target category, the scene could contain an example of the high-reward category as a task-irrelevant distractor (Fig. 3). Our core interest lay in these conditions, where we could isolate the neural response to an example of a nontarget object as a function of its prior reward association.

The scene stimuli used in the experiment are visually heterogeneous, and physical differences in the images and categories could drive variance in the neural response that might obscure effects of reward association. The experiment had two key features to control for this.

First, there were two groups of participants: one group associated reward with cars, the other with people. Second, plants were never associated with reward. Critical conditions were therefore those where participant groups were cued to search for the same neutral low-reward category (plants) and the scenes additionally contained a lateral example of either the high-reward distractor category or the low-reward distractor category (or both, with one presented laterally and the other centrally; see Figs. 1 and 3).

Critically, by manipulating the reward association across participant groups, we were able to use the same scenes in each of these experimental conditions. As an example of this, consider the trial illustrated in Figure 3*A*. For Group A, the high-reward target category is cars, but in this example the current target category is plants. The scene contains a single example of a person (alongside examples of the target). For participants in Group A, this scene therefore contains a lateral example of the low-reward distractor category. However, for participants in Group B, this same scene contains a lateral example of the high-reward distractor category. When results were collapsed across groups, physical differences in the scene stimuli were counterbalanced across participant groups.

In analysis, we use machine learning of EEG data to measure the quality of encoding and representation of reward-associated and neutral distractors, subsequently unpacking classification results through consideration of ERPs. In ERP analysis, our focus lay particularly on the N2pc (Luck and Hillyard, 1994) and Pd components (Hickey et al., 2009) as indices of attentional selection and suppression, respectively. To foreshadow, classification and ERP results demonstrate that naturalistic reward-associated distractors are strongly suppressed from the moment they appear.

### Participants

Thirty-six healthy volunteers from the University of Birmingham community gave informed consent before completing the experiment. Each participant reported normal or corrected-to-normal vision and was paid for participation. Two participants were rejected from analysis because of poor accuracy in low-reward task conditions (>2.5 SDs from the mean), leading to a final sample of 34. Of these, 3 were left-handed, 10 were men, and mean age was 20 years (3 years SD).

### Stimuli and procedure

Participants searched through black-and-white photographs of real-world scenes (∼22° × 17° visual angle) for examples of three different object categories. Figure 1 presents a set of scene examples. The target category changed for each of the 24 experimental blocks, with each block containing 54 trials, and a cue at the beginning of each block identified whether cars, people, or plants were the target category for that set of trials. When participants were cued to search for cars or people, every scene in the block contained a single example of the target category located at the left, middle, or right of the image. Participants were asked to report the facing direction of the target (e.g., if the car faced the left or the right) via button press with the left or right hand on a standard computer keyboard. When participants were cued to search for plants, the scenes contained at least one example of a tree or a bush, but not examples of both, and these were located anywhere in the scene. Participants reported whether the scene contained trees or bushes with a corresponding left- or right-hand keyboard response. The target category for each individual block was selected at random with the constraint that each category served as target for an equal number of blocks in the experiment.

The scenes could contain examples of the categories not currently acting as target. For example, when search was for people, the scene could contain examples of cars and plants as task-irrelevant nontargets. When these distractors were cars or people, only a single example appeared and was located at the left, middle, or right of the scene. When the distractors were plants, multiple examples could appear at any location.

When people or cars were the target category, scenes were constrained such that they contained either a lateral example of the target category, a lateral example of the target category and a central example of the other localized distractor category, or a lateral example of the localized distractor category and a central example of the target category (see Fig. 1). Equal numbers of these target lateral, target lateral/distractor central, and distractor lateral/target central scenes were presented. In each of these layouts, the facing direction of the target and the facing direction of the localized distractor were counterbalanced across images within each category, as was the presence of trees or bushes.

When plants were the target category, scenes were constrained such that they contained either a lateral example of a car distractor, a lateral example of a car distractor and a central example of a person distractor, a lateral example of a person distractor, or a lateral example of a person distractor and a central example of a car distractor. Equal numbers of these lateral distractor and lateral distractor/central distractor scenes were presented to the participant, and the facing direction of the distractors was counterbalanced across images within each category.

There were 304 scene images in the stimuli set, most taken from a set of 480 images used in an earlier publication (Hickey et al., 2019) with additional scenes generated using a digital camera. Each core image set (e.g., left-located left-facing car, central right-facing person, bush) had 4-8 individual examples. Examples from each core image set were used in the experiment in random order until all images in the set had been presented, at which point this process reset in new random order. The scene images were prepared such that the category example in the periphery was roughly equidistant from fixation in each image and such that people and vehicles had roughly consistent size across the image set.

In each trial, correct response was rewarded with points that had cash value, with the magnitude of reward varying as a function of target category. For 17 of the participants, correct responses to car targets resulted in high-magnitude reward (100 points), whereas correct responses to people or plant targets resulted in low-magnitude reward (1 point). For the remaining participants, correct responses to people resulted in high-magnitude reward, with cars and plants associated with low-magnitude reward. The points putatively determined a final pay range of £18 to £24, and participants were instructed to maximize points and therefore earnings, but at the end of the experiment total earnings were rounded to £24 for all participants.

The experiment took place in a dimly lit room, and participants were seated at ∼1 m distance from a 24 inch LED monitor (100 Hz refresh rate). As illustrated in Figure 2, each experimental block began with a 2 s cue indicating the target category for that block and a reminder of the high-reward category. Each trial began with presentation of a fixation cross for 250-750 ms (randomly selected from a uniform distribution) followed by presentation of a scene for 200 ms. The scene was subsequently replaced by a fixation cross until either the participant responded via keyboard button press or 1750 ms had passed. Reward feedback was then presented for 1000 ms, after which a new trial began. Feedback regarding task accuracy and speed was provided at the end of every experimental block and the session took ∼2.5 h, reflecting 1.5 h of experimental participation and 1 h of preparation and debriefing. Stimuli presentation relied on PsychToolbox-3 for MATLAB (Brainard, 1997).

### EEG recording and preprocessing

EEG was recorded at 1 kHz from 64 Ag/AgCl electrodes mounted in an elastic cap using a Biosemi Active2 amplifier and ActiView acquisition software. Horizontal electro-oculogram was recorded from electrodes 1 cm lateral the left and right external canthi, vertical electro-oculogram was recorded from electrodes place directly above and below the left pupil, and two additional electrodes recorded voltage over the left and right mastoid processes. Electrode offset was minimized and stabilized before the start of recording. EEG was acquired at DC with a 208 Hz anti-aliasing filter, resampled offline at 512 Hz, rereferenced to the average of mastoid signals, and bandpass filtered with a Hamming windowed FIR kernel (0.1-45 Hz; –6 dB at 0.05 Hz and 45.05 Hz). Epochs beginning 1 s before and ending 2 s after each scene onset were extracted from the data.

Infomax independent component analysis (Bell and Sejnowski, 1995) was used to identify variance stemming from ocular artifacts in the epoched data. The independent components representing horizontal and vertical eye movements were used to identify trials in which eye movements were made in the 600 ms interval following stimulus onset. Participants moved their eyes in 6%-18% of trials, and these were removed from further analysis. Components representing eye and muscle artifacts were subsequently removed from the data, as were trials resulting in incorrect response, and epoched data were baselined on the 200 ms interval preceding scene onset. Experimental conditions were subsequently defined based on the reward association of the target category (reward-associated car/person, neutral car/person, or neutral plant) and the presence and location of distractor stimuli.

### EEG machine learning classification

Our approach to EEG classification is based on linear discriminant analysis and cross-fold validation. Each classification analysis interrogates a conditional difference, for example, whether a target is located on the left or right of the scene, with the classifier trained to label data as coming from one of these two classes. In each analysis, conditional EEG is partitioned into 10 folds, each balanced to contain an equal number of randomly selected, correctly performed trials from each of the two classes, and a model is built for each combination of 9 data folds. The 10 resulting models, each based on a unique combination of 9 of 10 data folds, are subsequently tested against the individual trials contained in the single fold that did not contribute to model building. There is no trial averaging in our approach, and classification accuracy is defined as the mean testing accuracy across trials and folds. To establish a time course of classification accuracy, we implemented this modeling and validation procedure for each ∼2 ms sample point in an epoch beginning 250 ms before the onset of the scene stimulus and ending 1000 ms after. To ensure model stability and accuracy, models were built and tested on data spanning a 61-sample interval centered on the data point under consideration (constituting 64 × 61 = 3904 observations of electrode voltage). Each data point in classification analyses thus reflects classification performance across a ∼120 ms interval centered on the data point under consideration. This importantly means that the absolute latency of classification onset should be interpreted with care, as accuracy at a given time point reflects the performance of a model with access to data recorded up to ∼60 ms later. In contrast, peak classification latency and conditional effects on classification latency can be unambiguously interpreted.

To gain insight on model classification decisions, we extracted model weights in each model building instance. These were subsequently multiplied by the covariance matrix of the data that had been used to build the model, with the results mean averaged across model building iterations and across latency intervals of interest and *z*-scored within each participant before being mean averaged across participants. This procedure transforms the backward model generated by linear discriminant analysis, which projects a data pattern into an expected class membership, into a forward model, which projects class membership into an expected data pattern (Haufe et al., 2014). The forward model can be topographically plotted to illustrate the classifier decision criteria (see Fig. 6).

Statistical analysis of classification accuracy relied on threshold-free cluster enhancement (Smith and Nichols, 2009) with clusters defined over time. Conditional differences in classification accuracy were tested using permutation contrasts with 100,000 iterations based on mean accuracy observed in a 40 ms interval centered on the cross-conditional accuracy peak. Statistical analysis of the latency of classification accuracy relied on a resampling approach. To assess the difference in peak classification latency between conditions, we iteratively resampled from the set of 34 participant datasets 100,000 times with replacement. In each iteration, we averaged classification accuracy for the relevant conditions across the sample, extracted the peak latency for each condition, and calculated the difference in peak latencies. The probability that an observed difference in peak classification latency might have been observed under the null hypothesis was reflected in the proportion of the distribution of difference scores that fell below zero. Classification analyses relied on the COSMOMVPA (Oosterhof et al., 2016) and ADAM toolboxes (Fahrenfort et al., 2018) and on custom code.

### ERPs

ERPs were calculated using standard signal-averaging (Luck, 2014). Our focus was on the N2pc and Pd components of the visual ERP, which index attentional selection and attentional suppression, respectively, and emerge in visual cortex contralateral to the location of the eliciting stimulus. To isolate these components from bilateral variance in the ERP, we (1) extracted voltage recorded at electrodes located over left visual cortex when the eliciting stimulus was in the right visual field, and averaged this response with (2) voltage recorded at electrodes located over right visual cortex when the eliciting stimulus was in the left visual field. This generated a contralateral waveform, and a similar procedure was applied to generate ipsilateral waveforms. The contralateral and ipsilateral waveforms were calculated as the mean average of a cluster of three lateral occipital electrodes that are identified by larger marker in topographies included in the figures. Topographic maps of differences in lateralized ERP components are generated by “flipping” EEG data observed when the eliciting stimulus is in the right visual field and averaging with EEG data observed when it is in the left visual field, such that the left cortical hemisphere consistently represents ipsilateral cortex and the right cortical hemisphere consistently represents contralateral cortex.

Importantly, when calculated in reference to the objects appearing to the left and right of fixation, the N2pc and Pd are insensitive to lateralized activity evoked by objects in the center of the visual field (Woodman and Luck, 2003; Hickey et al., 2006, 2009). For example, consider a display with a central car distractor and a lateral person target, with the central distractor eliciting theoretical right-lateralized ERP activity. When the person target is in the left visual field, the car-elicited effect emerges as positivity in the contralateral signal. But when the person distractor is in the right visual field, the car-elicited effect emerges as negativity in the contralateral signal. When mean target-elicited contralateral signal is calculated, the central distractor has no summed effect.

Statistical analysis of ERP component amplitude depended on parametric repeated-measures ANOVA. Lateral ERPs were statistically assessed in two latency intervals: 220-280 ms, when the N2pc and Pd are known to emerge with maximum amplitude (Luck and Hillyard, 1994; Hickey et al., 2009); and 100-160 ms, when an early expression of the Pd is known to emerge (Sawaki and Luck, 2010; Weaver et al., 2017). ERP analysis relied on the EEGLAB toolbox (Delorme and Makeig, 2004) and custom code. Additional control analyses involving linear mixed models and Bayesian model comparison are described in Results and depend on the *fitlme.m* function implemented in the MATLAB statistic toolbox (R2021b) and the BayesFactor toolbox (https://klabhub.github.io/bayesFactor) with default priors.

## Results

### Behavior

**Figure 4.**
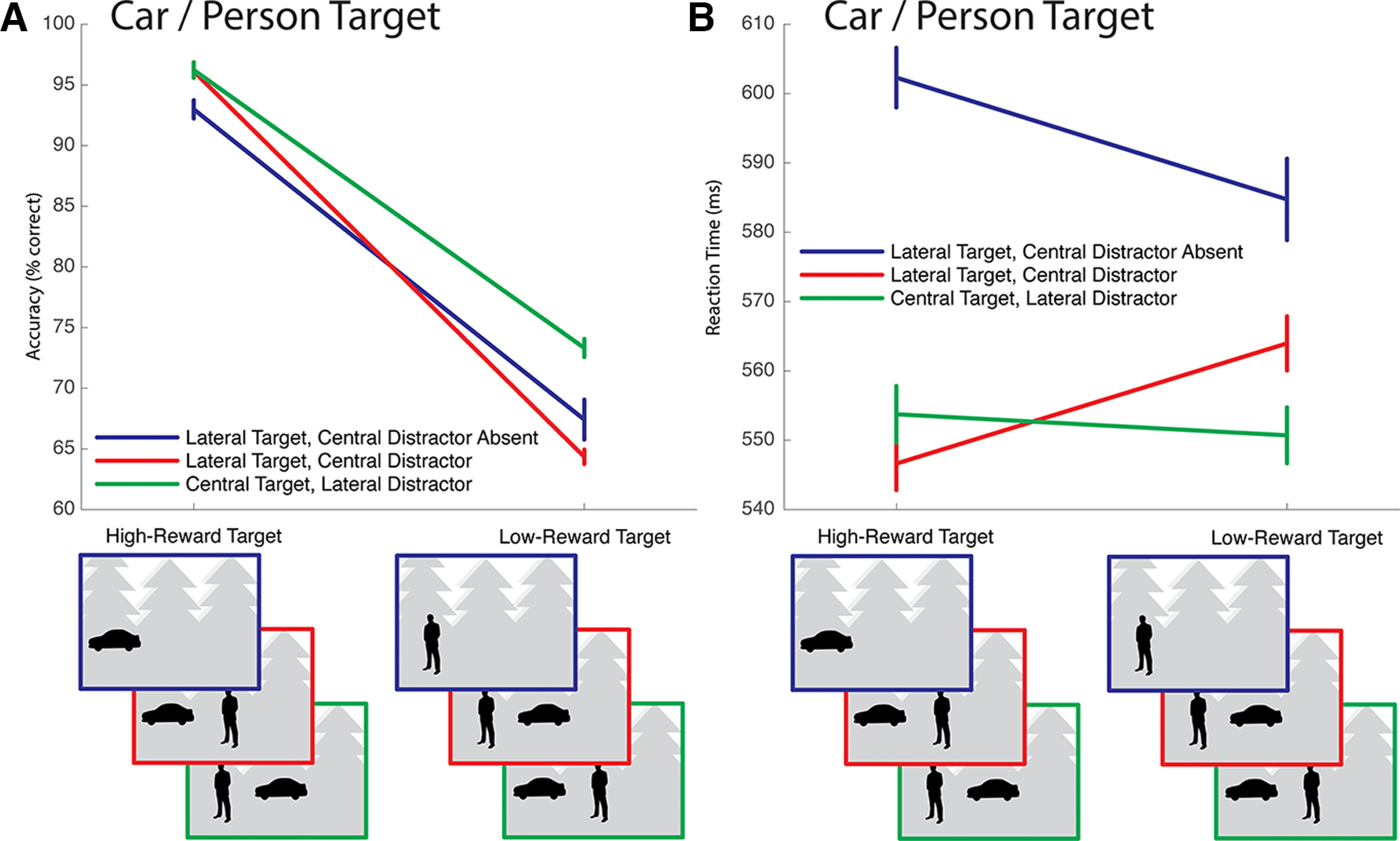
Behavioral results from conditions where cars or people were the target of search. These results are presented for the sake of completeness; no core hypotheses are tested. In the stylized scene examples used here and in subsequent figures, high-magnitude reward is associated to cars; but as described in the body of the paper, this was counterbalanced across participants. ***A***, Accuracy. As expected, responses to high-reward targets were more accurate in all conditions. ***B***, Reaction times. Surprisingly, participants were faster to respond to targets presented in scenes that also contained an example of the localized distractor. This may reflect a qualitative difference in the images; scenes containing only one localized category type happened to be characterized by smaller, harder-to-find target examples. Data collected from presentation of these scenes were not used to test the core study hypothesis regarding the capture of attention to reward-associated stimuli.

**Figure 5.**
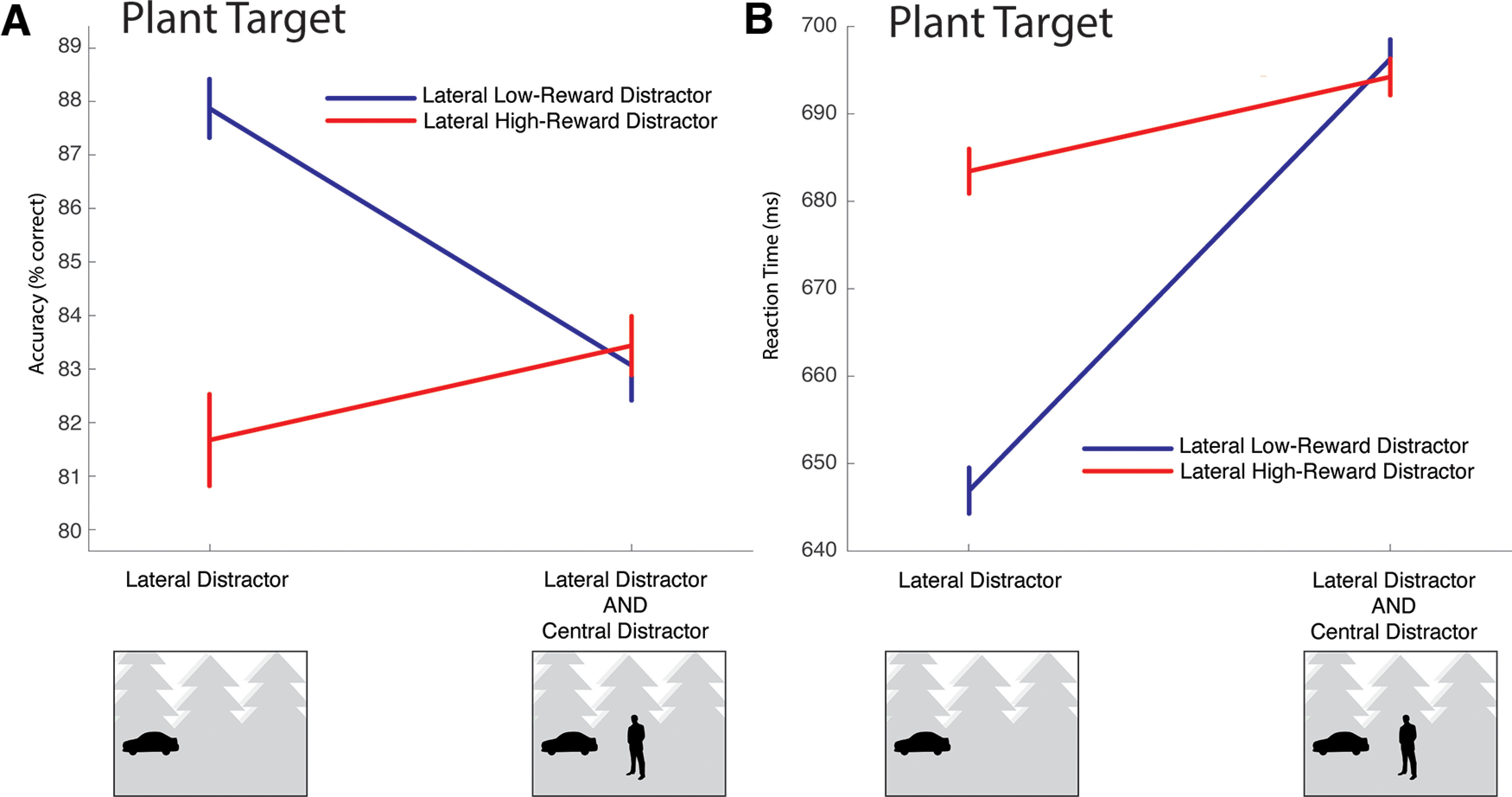
Results from conditions where participants searched for plants, which were used to test motivating experimental hypotheses regarding attentional capture. ***A***, When participants searched for plants in scenes containing a single additional distractor, accuracy degraded when that distractor was associated with reward. However, when the scene contained both a lateral and a central distractor — and therefore always contained examples of both the high-reward and low-reward distractor categories — accuracy was insensitive to the specific locations of the two distractors. ***B***, Similar results emerge in reaction times. When participants searched for plants in scenes containing a single additional distractor, reaction times increased when that distractor was associated with reward. However, when the scene contained two distractors, reaction time was insensitive to the specific location of these distractors.

Outliers were defined as responses where reaction time was >3 SDs from the participant mean and were rejected from further analysis (1.3% of trials, 0.4% SD). Accuracy and reaction time are illustrated in Figures 4 and 5. The results illustrated in Figure 4*A*, *B* are presented largely for the sake of completeness as no critical experimental hypotheses are tested. As expected, participants were more accurate when correct response to the target garnered high-magnitude reward (Fig. 4*A*). Unexpectedly, they were slower to respond to the target when the distractor was absent from the scene (Fig. 4*B*).

**Figure 6.**
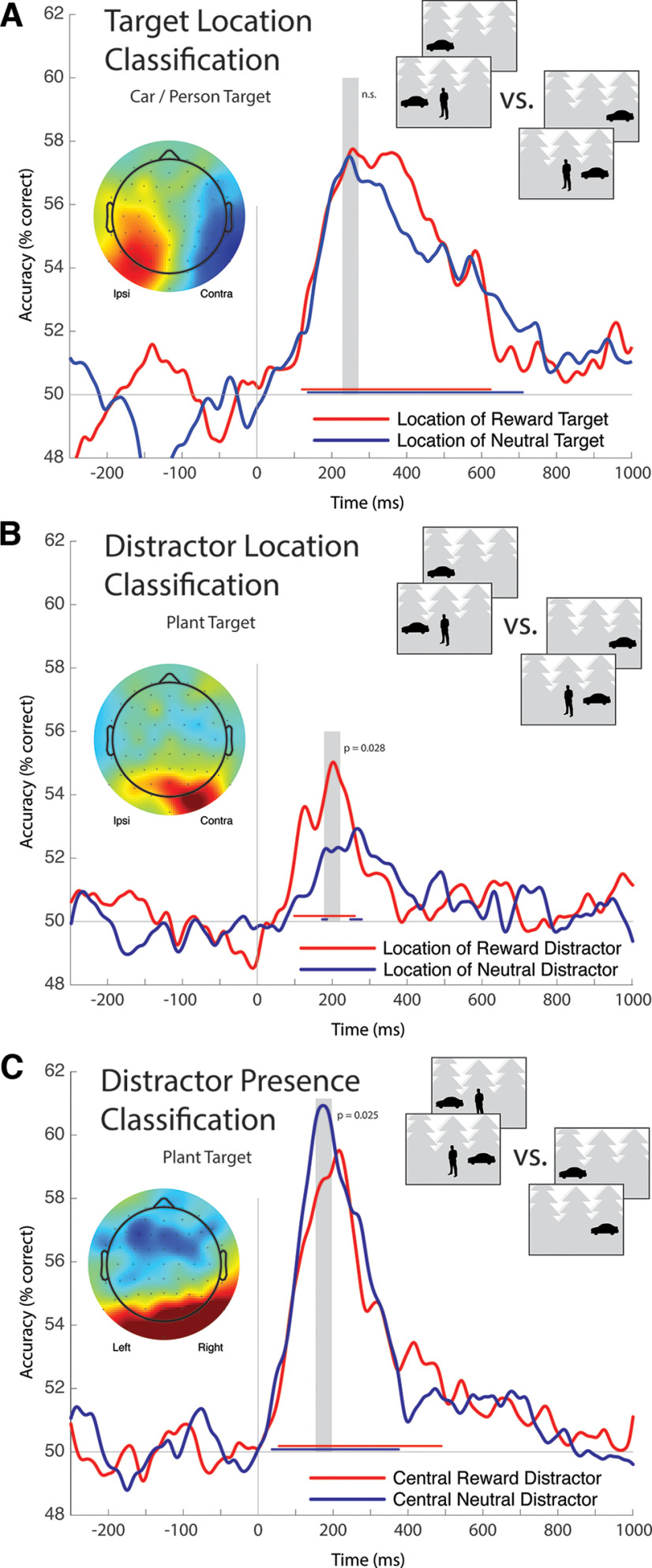
Results from trial-wise EEG classification. In all analyses, the machine learning algorithm is trained to discriminate between two classes of stimuli, and 50% accuracy reflects chance performance. Topographic maps reflect model decision criteria across reward conditions as measured over the latency intervals identified by gray shading in the corresponding time course plots, which is a 40 ms interval centered on peak decoding accuracy collapsed across conditions. Topographic plots are computed as the mean of spatially *z*-scored data for each individual and are therefore in SD units; while the topographical pattern is informative, the underlying values are uninformative and, as such, no scale is provided in the figure. Significant classifier accuracy in each condition is illustrated in the red or blue lines located just above the 50% baseline. ***A***, Results from classification of target location. This analysis is intended to demonstrate the efficacy of the method, and to investigate the impact of reward association on target processing, but does not test the motivating experimental hypothesis regarding the capture of attention. Analysis does not identify a reliable difference in classification accuracy as a function of target reward association. ***B***, Results from classification of distractor location. Location classification improves when the distractor is associated with reward. ***C***, Results from classification of whether the distractor was present in the center of the scene. Presence classification degrades when the distractor is associated with reward.

Results from the critical plant target condition are presented in Figure 5. When a lateral distractor was present in the scene during search for a plant, the association of reward to the distractor decreased accuracy (Fig. 5*A*, left) and increased reaction time (Fig. 5*B*, left). When the scene contained both a lateral distractor and a central distractor — and therefore contained both a reward-associated and a neutral distractor — the specific location of the reward-associated and neutral distractors did not have an impact on accuracy (Fig. 5*A*, right) or reaction time (Fig. 5*B*, right).

Statistical analysis of accuracy in the plant target condition took the form of a repeated-measures ANOVA with factors for group (cars are reward-associated vs people are reward-associated), reward association of lateralized distractor (reward-associated lateral distractor vs neutral lateral distractor), and presence of central distractor (central distractor present vs central distractor absent). This identified a main effect of distractor reward association (*F*_(1,32)_ = 18.68, *p* < 0.001, *η^2^_p_* = 0.369), reflecting the decrease in accuracy when a reward-associated distractor was present, and an interaction of distractor reward association and central distractor presence (*F*_(1,32)_ = 22.19, *p* < 0.001, *η^2^_p_* = 0.410), reflecting accentuation of this effect when the central distractor was absent from the scene. No other effects reached significance (central distractor presence: *F*_(1,32)_ = 3.09, *p* = 0.088; group × distractor reward association: *F*_(1,32)_ = 2.91, *p* = 0.098; group × central distractor presence: *F*_(1,32)_ = 2.66, *p* = 0.113; three-way interaction: *F*_(1,32)_ = 1.74, *p* = 0.196; all other *F* values < 1).

A similar pattern of results emerged from analysis of reaction time. A repeated-measures ANOVA with the same factors identified a main effect of distractor reward association (*F*_(1,32)_ = 41.30, *p* < 0.001, *η^2^_p_* = 0.563) and a main effect of central distractor presence (*F*_(1,32)_ = 94.84, *p* < 0.001, *η^2^_p_* = 0.747), alongside an interaction of distractor reward association and central distractor presence (*F*_(1,32)_ = 59.12, *p* < 0.001, *η^2^_p_* = 0.649). No other effects reached significance (group: *F*_(1,32)_ = 1.73, *p* = 0.198; all other *F* values < 1).

The critical observation from these behavioral results is that the reward-associated distractor decreased accuracy and increased reaction times relative to the neutral distractor. As we found no behavioral differences between the two subject groups, we collapse across this distinction in subsequent analysis of electrophysiological data.

### EEG classification

As illustrated in Figure 6, we conducted three independent classification analyses of EEG data. The first was focused on trials where participants searched for cars or people, separating these into conditions based on the location of the target (left hemifield vs right hemifield) and the reward association of the target (reward-associated target vs neutral target). This analysis did not directly test our motivating hypothesis regarding attentional capture to distractor stimuli, but it allowed us to characterize how classification of target location emerged in EEG data and describe the impact of reward association on target processing. Classifiers were trained to identify the location of the lateral target for each of the reward-associated and neutral target conditions separately. As presented in Figure 6*A*, classification accuracy at the cross-conditional peak (231-271 ms) did not reliably differ as a function of the target reward association (*p* = 0.440). Accuracy subsequently diverges between conditions, but this difference did not survive cluster correction for multiple comparisons.

The topographic map presented in Figure 6*A* illustrates a forward projection of the classification model collapsed across reward-associated and neutral target conditions and averaged over the 231-271 ms interval identified by gray shading in the figure. The scalp map shows a lateral pattern, with the model classifying a trial as containing a target in the left hemifield when right posterior cortex had more negative voltage, and vice versa. This suggests that the model is loading on variance that also underlies the N2pc, as has been observed in earlier classification analysis of EEG from visual search (Fahrenfort et al., 2017). Results from this classification analysis appear to reach a ceiling, with reward association not causing the EEG signal to carry additional information about the target location.

The second classification analysis is analogous to that described above but focused on classification of distractor location and limited to trials where participants searched for plants. This analysis tests the motivating idea for the study, namely, that reward association might impact neural responses to distractor stimuli indexing attentional selection. As illustrated in Figure 6*B*, distractor location classification emerged quickly and showed a marked difference as a function of whether the distractor category had been associated with reward in prior experience. Across an interval centered on the cross-conditional classification peak (181-221 ms), accuracy was significantly greater when the distractor was taken from the reward-associated stimulus category rather than the neutral stimulus category (*p* = 0.028). The EEG signal thus carried more information about the location of the reward-associated distractor than it did about the location of the neutral distractor.

At first blush, this pattern is consistent with the idea that attention is captured by examples of the reward-associated distractor category. This mis-deployment of attention could cause the EEG signal to carry more information about the distractor location, and this could be associated with the degradation of overt response to the target. However, consideration of the topographic maps illustrated in Figure 6*A*, *B* identifies an inconsistency in this account. The forward projection of the classification model for distractor localization shows that the model classified a trial as containing a distractor in the left visual hemifield when signal over right posterior cortex had voltage more positive than that over left posterior cortex, and vice versa. This contrasts with results from classification of target location, where contralateral negativity, not positivity, contributed to the model decision.

To probe this disparity, we conducted an additional analysis to classify whether a scene contained a central distractor. This was again based on data collected while participants searched for plants, but rather than classifying distractor location, the model labeled trials as either containing a distractor in the center of the photograph, or not. As illustrated in Figure 6*C*, the machine learning algorithm was able to perform this task well, with cross-conditional peak decoding accuracy emerging at 176 ms. Topographic projection of the forward model suggests that the classification decision depended on emergence of brain activity in both visual cortex and frontocentral cortex. Classification accuracy was better when the central distractor had been previously associated with neutral outcome than with reward outcome (156-196 ms interval; *p* = 0.025).

There is the possibility that distractor location classification and distractor presence classification are related to one another. That is, the contralateral positivity that emerges in classification of distractor location might reflect activation of a mechanism in visual cortex that also emerges when a distractor is present in the center of the scene. Consistent with this, classification of distractor presence appears to depend on bilateral posterior positivity (Fig. 6*C*). However, reward association has a positive impact on distractor location classification, but a negative effect on distractor presence classification, and this pattern is hard to explain if classification in both instances is associated with the same EEG variance.

An alternative account for this pattern — better classification of the location of a reward-associated distractor, but poorer identification of the presence of a reward-associated distractor — is that the reward-associated distractor is suppressed in the poststimulus interval. Under this premise, classification of the reward-associated distractor is more accurate because this stimulus triggers a response in contralateral visual cortex (a Pd) that acts to inhibit encoding of this stimulus. The machine learning algorithm uses this index of visual suppression to infer distractor location, but separate classification of stimulus presence is poor because this suppression leads to a degraded encoding of the stimulus and its associated category.

We conducted two additional analyses of classification accuracy to further test this interpretation. In the first, we examined the latency of peak decoding accuracy for distractor location and distractor presence separately for reward-associated and neutral distractors. Peak classification of the presence of a reward-associated distractor emerged at roughly the same latency as peak classification of the location of a reward-associated distractor (209 ms vs 195 ms; −14 ms difference). However, peak classification of the presence of a neutral distractor preceded peak classification of its location (176 ms vs 264 ms; 89 ms difference). Resampling statistics based on these difference scores suggested that the difference in latencies observed for the neutral distractor reliably differed from those observed for the reward-associated distractor (*p* = 0.038), with follow-up contrasts failing to identify a latency difference between location classification and presence classification for reward-associated distractors (*p* = 0.276) but identifying a marginal trend for neutral distractors (*p* = 0.061). This suggests that the EEG signal may carry information about the presence of the neutral distractor that precedes information about its location, consistent with classic theoretical perspectives proposing that diagnostic feature information is extracted from visual input before being localized (Treisman and Gelade, 1980; Wolfe et al., 1989). This does not occur for the reward-associated distractor, in line with the idea that brain activity underlying location classification leads to a degraded representation of the distractor, and therefore poorer classification of object presence.

**Figure 7.**
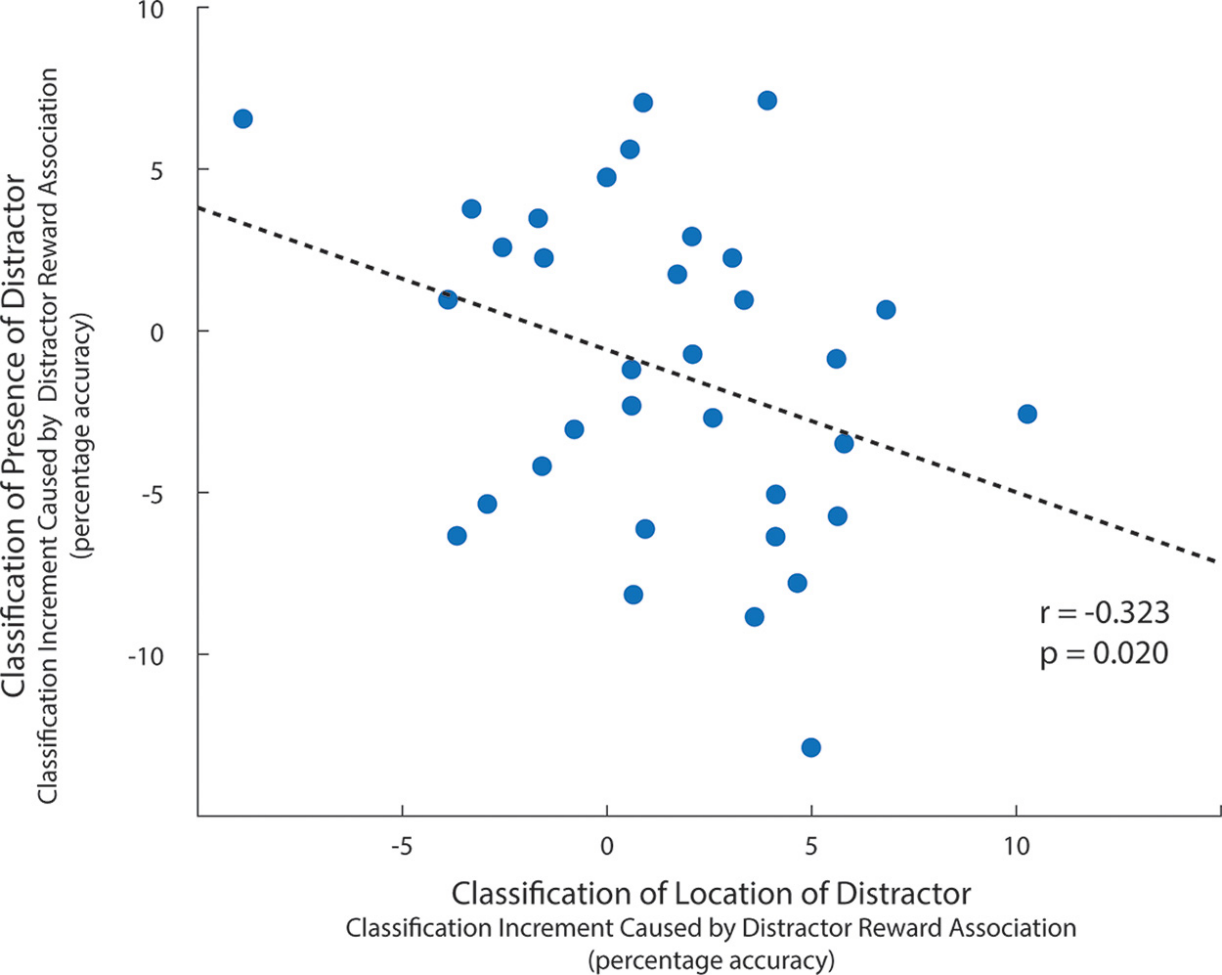
Relationship between classification of distractor location and distractor presence across participants. Statistical analysis reflects permutation analysis with 100,000 iterations, an approach that is robust to the influence of outliers.

In the second analysis, we tested the relationship between the effect of reward on distractor location classification and presence classification. If the EEG variance that supports location classification causes poor classification of distractor presence, there should be a negative relationship between these effects across the experimental sample. To this end, we extracted average location and presence classification accuracy across a 100-300 ms latency range for each of the high-reward and low-reward conditions for each of the 34 participants. This interval includes latencies where mechanisms of target processing and distractor suppression are known to emerge in the EEG and ERP (Luck and Hillyard, 1994; Hickey et al., 2009; Weaver et al., 2017). As illustrated in Figure 7, as classification accuracy for high-reward distractors increased across individuals (relative to low-reward distractors), presence classification decreased (relative to low-reward distractors).

### ERPs

Classification of target location appears to rely on emergence of contralateral negativity in posterior cortex, suggesting it is driven by variance underlying the N2pc, whereas classification of distractor location appears to rely on emergence of contralateral positivity, suggesting it is driven by the Pd. However, results from classification leave some ambiguity regarding the contribution of these posterior effects to classification. It is particularly unclear whether the difference in distractor location classification as a function of reward association is driven by the Pd or other sources of variance in the EEG signal.

**Figure 8.**
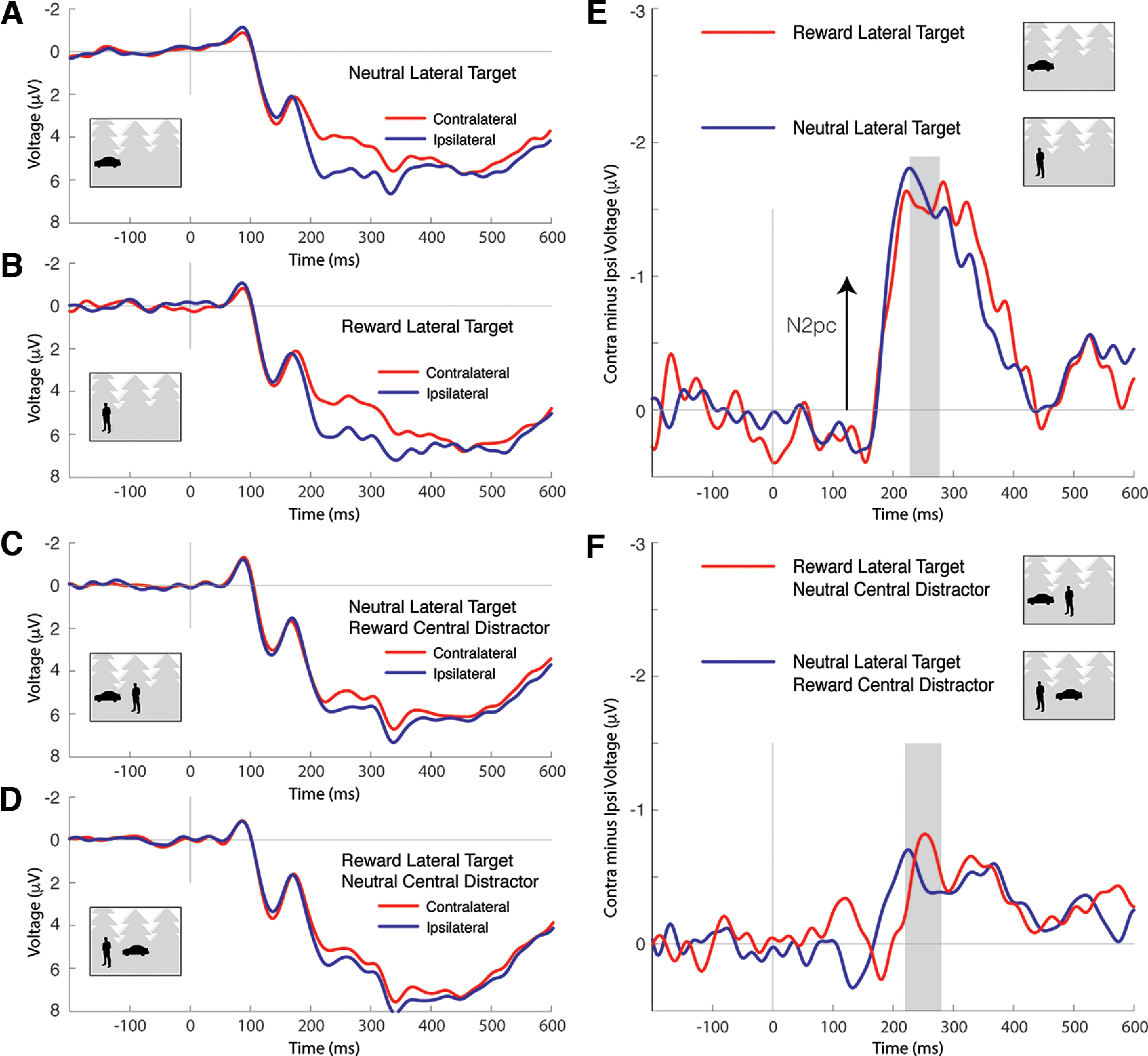
Target-elicited ERPs in car/person target conditions. In the stylized stimulus examples here and in Figure 7, the reward-associated category is cars, but this was counterbalanced across participants in the actual experiment. Here and in Figure 7, negative voltage is plotted upward by convention and the ERPs reflect mean signal observed at the lateral electrode clusters identified by large marker in the topographic maps in Figure 7. ***A***, The posterior lateral ERPs elicited by a scene containing a peripheral neutral target. The N2pc is apparent as the difference between contralateral and ipsilateral waveforms beginning at ∼200 ms after stimulus. ***B***, The ERPs elicited by a scene containing a peripheral reward-associated target. ***C***, The ERPs elicited by a scene containing a peripheral neutral target when a task-irrelevant example of the reward-associated category is present in the center of the scene. ***D***, The ERPs elicited by a scene containing a peripheral reward-associated target when a task-irrelevant example of the neutral target category is present in the center of the scene. ***E***, Contralateral-minus-ipsilateral difference waves for the ERPs illustrated in ***A***, ***B***. The N2pc is reflected in negative deflection of the difference wave and does not reliably differ as a function of target reward association. ***F***, Difference waves for the ERPs illustrated in ***C***, ***D***. As in ***E***, the N2pc does not reliably differ as a function of target reward association.

To address this ambiguity, and to generally unpack the classification results, we extracted ERPs from the experimental data and isolated the N2pc and Pd components. Figure 8 illustrates ERP results when the target of search was a car or person and the target appeared at a lateral location. These analyses do not test our core motivating hypothesis regarding the capture of attention to reward-associated distractors, but, as with classification of target location, allow us to additionally characterize how reward association impacted target processing and to identify the relationship between ERP results and classification. Lateral waveforms are presented in Figure 8*A–D*, and contralateral-minus-ipsilateral difference waves are presented in Figure 8*E*, *F*. When the lateral target was presented without a central distractor, it elicited a robust N2pc that did not reliably vary as a function of the manipulation of reward outcome (Fig. 8*E*). A smaller N2pc was elicited by the lateral target when a central distractor was present in the scene, reflecting the distracting effect of a prominent foreground nontarget at fixation, but, again, the N2pc did not show a reliable effect of target reward association (Fig. 8*F*). The N2pc results are thus very similar to the pattern observed in classification accuracy (Fig. 4*A*). In line with this, the N2pc elicited by a lateral target reliably correlates with target location classification across participants (mean 200-300 ms, *r* = 0.273, *p* = 0.028, permutation test with 100,000 iterations).

To statistically assess the pattern of results in target-elicited N2pc, we conducted a repeated-measures ANOVA based on mean ERP amplitude from 220 to 280 ms, a latency interval where the N2pc is known to be maximal (Luck and Hillyard, 1994) and where it emerged prominently in the current data. The repeated-measures ANOVA had factors for electrode laterality (ipsilateral vs contralateral), target reward association (reward vs neutral), and central distractor presence (present vs absent), and identified main effects of electrode laterality (*F*_(1,33)_ = 73.74, *p* < 0.001, *η^2^_p_* = 0.691), reflecting consistent emergence of N2pc across conditions, and central distractor presence (*F*_(1,33)_ = 5.15, *p* = 0.030, *η^2^_p_* = 0.135), reflecting a positive shift in the bilateral ERP when the distractor was present. Electrode laterality interacted with distractor presence (*F*_(1,33)_ = 33.49, *p* < 0.001, *η^2^_p_* = 0.504), reflecting the increase in N2pc amplitude in the distractor absent condition, but no other interactions emerged (reward × distractor presence: *F*_(1,33)_ = 1.34, *p* = 0.255; all other *F* values < 1).

**Figure 9.**
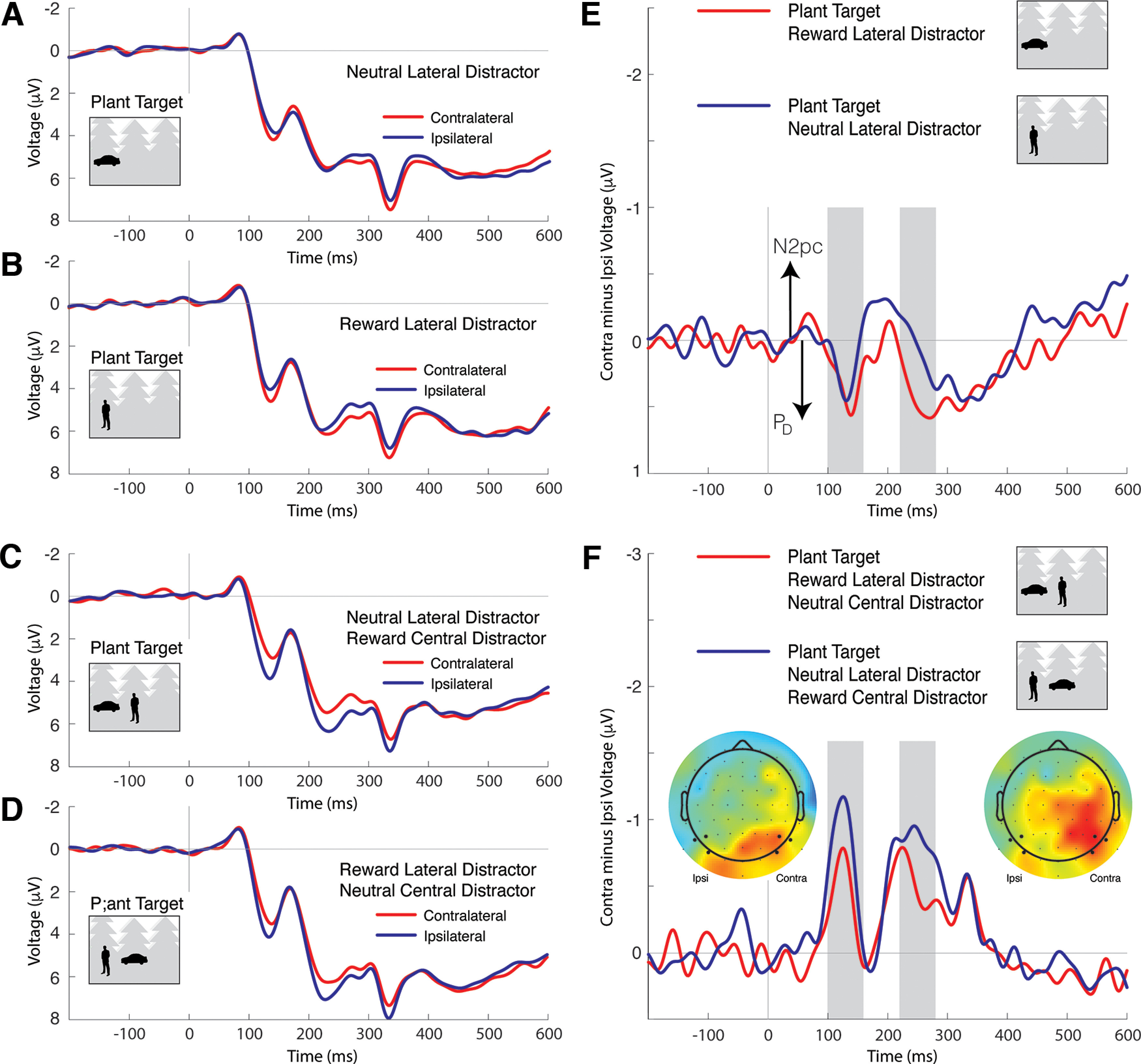
Distractor-elicited ERPs in plant target conditions. ***A***, The posterior lateral ERPs elicited by a scene containing a peripheral neutral distractor. ***B***, The ERPs elicited by a scene containing a peripheral reward-associated distractor. ***C***, The ERPs elicited by a scene containing a peripheral neutral distractor when a reward-associated distractor is present in the center of the scene. ***D***, The ERPs elicited by a scene containing a peripheral reward-associated distractor when a neutral distractor is present in the center of the scene. ***E***, Contralateral-minus-ipsilateral difference waves for the ERPs illustrated in ***A***, ***B***. The N2pc is reflected in negative deflection of the difference wave, and the Pd is reflected in positive deflection. ***F***, Difference waves for the ERPs illustrated in ***C***, ***D***. Topographic maps reflect the conditional difference in voltage observed in the intervals identified by gray shading in the difference waves. The electrodes included in the calculation of ipsilateral and contralateral waveforms are identified by larger marker.

Figure 9 illustrates ERP results when search was for plants and a reward-associated or neutral distractor appeared at a lateral location. Results from these critical experimental conditions directly address motivating hypotheses regarding the capture of attention to reward-related distractors. Lateral waveforms are presented in Figure 9*A–D*, and contralateral-minus-ipsilateral difference waves are presented in Figure 9*E*, *F*. When the lateral distractor appeared without a central distractor, it elicited a lateral response that had both positive and negative components (Fig. 9*E*). This may reflect the imbalance in sensory energy in these scenes; the lateral distractor is a prominent foreground object in these photographs without a corresponding object in the contralateral field, and this imbalance in sensory stimulation may have elicited contralateral activity in visual cortex linked to sensory and perceptual processing unrelated to the deployment of attention. The important observation is that the lateral ERP consistently has more positive polarity in the latency of N2pc and Pd when the eliciting distractor has been associated with reward. This is the case both when the scene contains a central distractor (Fig. 9*F*) and when it does not (Fig. 9*E*). This relative positivity in the lateral ERP suggests that selection of the reward-associated distractor was degraded, relative to selection of the neutral distractor.

We statistically assessed this pattern in two latency intervals: 100-160 ms, which corresponds to the interval when early Pd emerges (Sawaki and Luck, 2010; Weaver et al., 2017), and 220-280 ms, when the N2pc and Pd are maximal (Luck and Hillyard, 1994; Hickey et al., 2009). In both cases, repeated-measures ANOVA had factors for electrode laterality (ipsilateral vs contralateral), distractor reward association (reward vs neutral), and central distractor presence (present vs absent). Results from the early latency period identify a single main effect of distractor presence (*F*_(1,33)_ = 11.65, *p* = 0.002, *η^2^_p_* = 0.261) alongside an interaction of electrode laterality and distractor reward association (*F*_(1,33)_ = 4.32, *p* = 0.046, *η^2^_p_* = 0.116), reflecting the positive shift in the lateral waveform elicited by a reward-associated distractor, and an interaction of electrode laterality and distractor presence (*F*_(1,33)_ = 15.48, *p* < 0.001, *η^2^_p_* = 0.319), reflecting the negative shift in the lateral waveform when the central distractor was present (electrode laterality: *F*_(1,33)_ = 3.80, *p* = 0.060; reward: *F*_(1,33)_ = 1.84, *p* = 0.184; all other *F* values < 1). Much the same pattern emerged in analysis of the later time window, with a main effect of electrode location (*F*_(1,33)_ = 4.13, *p* = 0.050, *η^2^_p_* = 0.111), an interaction of electrode location and reward (*F*_(1,33)_ = 6.23, *p* = 0.018, *η^2^_p_* = 0.159), an interaction of electrode location and distractor presence (*F*_(1,33)_ = 13.43, *p* < 0.001, *η^2^_p_* = 0.289), but no other effects (reward: *F*_(1,33)_ = 2.13, *p* = 0.154; distractor presence: *F*_(1,33)_ = 2.33, *p* = 0.134; all other *F* values < 1). To relate these ERP effects to classification, we extracted the contralateral signal in the distractor-elicited ERP, collapsing across reward conditions but focusing on the 100-300 ms interval where these effects emerged. Increase in positivity in the ERP in this interval reliably predicted an increase in distractor location classification accuracy across individuals (*r* = 0.304, *p* = 0.036, permutation test with 100,000 iterations). This supports the notion that distractor location classification loads on the Pd, as suggested above.

One possibility is that the accentuated suppression of high-reward distractors we identify in Figure 9 is a reaction to the deployment of attention to the reward-associated distractor. However, this hypothesis (i.e., deployment of attention to the distractor followed by distractor suppression) should express as an initial contralateral negativity followed by a contralateral positivity (e.g., Hickey et al., 2006; Sawaki and Luck, 2013). Instead, lateral positivity emerges very quickly, ∼40 ms after afferent activity reaches visual cortex, leaving little opportunity for preceding cognitive operations.

Another possibility is that the reward-associated distractor may initially draw attention within each block, but that participants learn to rapidly suppress this object as they gain experience, either strategically or through the influence of implicit statistical learning (Ferrante et al., 2018, 2023). This predicts that the difference in lateral response to neutral and reward-associated distractors should change over the 54 trials in a block. In the extreme case, the reward-associated distractor could initially elicit a contralateral negativity — indicative of attentional capture — but later a contralateral positivity, reflecting the establishment of inhibitory control.

To test this, we used linear modeling and Bayesian model comparison to assess the impact of trial position within an experimental block. We split observations from each block of trials into two sets: one describing observations from the first half of a block and the other describing those from the second half of a block. If the difference in lateral ERP elicited by reward-associated versus neutral distractors changes over the course of an experimental block, this should emerge as a three-way interaction of electrode location (ipsilateral vs contralateral), distractor reward association (reward vs neutral), and block position (first half of block vs second half of block). To measure the impact of this three-way interaction, we repeatedly built mixed linear models for each of the two latency intervals of interest. An initial full model included a random intercept for each experimental participant and fixed factors for reward, distractor presence, electrode location, block position, and all possible interactions between these factors. A restricted model included all these factors, except for the interaction of electrode location, reward, and block position. Bayesian statistics were used to compare the full and restricted models, generating Bayes factor values for each of 1000 iterated model instances that were subsequently mean averaged. Results from analysis of the early latency interval (100-160 ms) revealed moderate to strong evidence in favor of the null hypothesis of statistical equivalence of the full and restricted models (average Bayes factor = 0.127), and analysis of the late interval generated similar results (220-280 ms; average Bayes factor = 0.140). The difference in lateral response to reward-associated and neutral distractors, statistically expressed in the interaction of electrode position and reward, therefore appears insensitive to the position of a trial within an experimental block. This suggests that the stimulus-driven suppression indexed in Pd emerges quickly and does not require extended experience of the distractor category.

## Discussion

We tested the idea that examples of a reward-associated object category capture attention during search through photographs of real-world scenes. Participants searched for examples of a cued target category — cars, trees, or people — while we recorded electrical brain activity. Importantly, the scenes contained examples of the nontarget object categories as task-irrelevant distractors. One of the three object categories was associated with financial reward, and our interest lay in conditions where search was for a neutral target, but the scene happened to contain an example of the reward-associated category as a task-irrelevant distractor. Behavioral analysis shows that participants were slower and less accurate to respond to the target in this circumstance, compared with when the scene contained a neutral distractor. This behavioral pattern has two possible explanations: attention may be captured to the reward-associated distractor, or the reward-associated distractor may create filtering costs and the need for cognitive control. Results from EEG unambiguously show that the behavioral effect is not a reflection of attentional capture. Instead, it appears that the reward-associated distractor is suppressed almost immediately after the scene appears. This suppression is indexed in a shift in distractor-evoked brain activity toward contralateral positivity, indicative of emergence of the Pd component of the visual ERP (Hickey et al., 2009; van Zoest et al., 2021), and in degraded accuracy of machine learning classification of distractor presence.

These results contrast with those from existing EEG and MEG studies using synthetic visual search arrays, where reward-associated distractors appear to robustly capture attention. For example, Hickey et al. (2010) had participants search for a uniquely shaped target in an array of distractors, one of which had a unique color. When selection of the target resulted in high magnitude reward, and the target and salient distractor colors subsequently swapped between trials, the distractor captured attention and elicited a robust N2pc. Similarly, Qi et al. (2013) used a training paradigm to associate reward to a color. When the task changed, and color was rendered task-irrelevant, distractors characterized by the reward-associated color continued to capture attention and elicit an N2pc. In these and other studies, reward appears to impact the representation of the task-irrelevant target-characterizing feature such that stimuli with this feature capture attention.

Why do reward-associated synthetic distractors capture attention, but reward-associated naturalistic distractors do not? One important observation is that the locus of learning differs across these two contexts. In the studies of synthetic visual search described above, learning presumably involves relatively early visual cortex where low-level features are represented. In studies of naturalistic vision, by contrast, reward is associated to a visually heterogeneous category of real-world objects where category membership is not predicted by the presence of specific low-level visual features, and learning impacts encoding in ventral visual cortex, where mid-level features and visual semantics are represented (e.g., Hickey and Peelen, 2015, 2017). The association of reward to low-level features may lead to quicker and stronger effects on visual resolution. A second, related observation is that the simplified context of synthetic visual search may provide a better opportunity for incentive salience to cause attentional capture. Relative to naturalistic environments, synthetic search arrays contain only a small set of objects that are characterized by a limited set of nonoverlapping visual features. Reward-associated objects may become particularly prominent in this impoverished setting in a way that does not occur in the richer and more complicated context of real-world scenes. Finally, there is the possibility that these differences of perceptual complexity may impact how attentional mechanisms are recruited during search. It may be that the rapid suppression we observe here is only strategically recruited when the visual field contains perceptually complex information with strong competition for limited resources. In line with this, results have shown that reward-associated synthetic distractors are also suppressed in early visual cortex, but only when perceptual competition is high (Gong et al., 2017).

The idea that reward-associated naturalistic distractors draw attention, but do not necessarily capture it, is broadly in line with a deep literature in visual cognition centered on the idea of signal suppression (e.g., Folk and Remington, 1998; Leber and Egeth, 2006; Sawaki and Luck, 2010; Gaspar and McDonald, 2014; for review, see Gaspelin and Luck, 2019). The key proposal here is that salient stimuli may elicit representation in “salience maps,” but that signal at this stage of visual processing can be suppressed so that it does not impact the “priority maps” that ultimately determine how attention is deployed. For example, in Sawaki and Luck (2010), participants searched through arrays of letters for a target defined by combination of size and character. One of the nontarget letters was rendered salient by unique color, and results showed that these distractors elicited a prominent Pd component in the ERP, reflecting suppression. As in the current study, this Pd emerged from very soon after stimulus onset, leaving little opportunity for preceding attentional operations. The authors suggested that the salient distractor elicited a salience signal that drew attention to its location. However, because participants knew this salience signal would only identify task-irrelevant stimuli, they strategically inhibited this signal so that the underlying stimulus did not gain selective control (compare Bacon and Egeth, 1994; see also Sawaki and Luck, 2013; Drisdelle and Eimer, 2021; Stilwell et al., 2022). The current results suggest that this signal suppression hypothesis can be broadened to describe visual processing of stimuli rendered attention-drawing through reward association. In critical conditions, participants knew that stimuli characterized by incentive salience were task-irrelevant. They appear to have been able to establish control through suppression, stopping the deployment of spatial attention and limiting the encoding of information about the task-irrelevant object.

If the distractor is suppressed, why is its presence associated with a behavioral cost? Some studies of synthetic visual search find that emergence of distractor suppression is associated with an elimination of behavioral distractor costs (e.g., Sawaki and Luck, 2010; Gaspelin and Luck, 2019). However, it is more common to find that distractor suppression reduces distractor costs but does not eliminate them (e.g., Kiss et al., 2012; Jannati et al., 2013; Burra and Kerzel, 2014; Gaspar and McDonald, 2014). One account for this pattern is that the stimulus-triggered distractor suppression is inefficient. Models of visual attention suggest that the primary purpose of attentional suppression is to shelter neural representations of attended stimuli, limiting interference during the transformation of target information to decisions and behavior (e.g., Desimone and Duncan, 1995; Tsotsos et al., 1995; Luck et al., 1997). If stimulus-triggered suppression is delayed or inefficient, distractors may still interfere with ongoing cognition, though to a lesser degree. A complementary possibility is that residual distractor costs may not reflect interference at all, but rather the cognitive load of stimulus-triggered suppression. By this, effective implementation of stimulus-triggered distractor suppression, which must occur quickly following stimulus onset, may take time and resources, delaying or diminishing the deployment of attention to the target and in this way impacting the speed and accuracy of response (Treisman et al., 1983; Folk and Remington, 1998).

To date, the body of neuroscientific literature examining incentive salience in naturalistic visual search has largely relied on fMRI, showing that information about reward-associated distractor categories is degraded in ventral visual cortex (Seidl et al., 2012; Hickey and Peelen, 2015, 2017; Barbaro et al., 2017). This has been counterintuitively interpreted as evidence of the capture of attention to these stimuli. The logic here is that capture will be quick and followed by longer-lived suppression of the distractor to allow search for the target to continue. The notoriously poor temporal resolution of fMRI means that any accentuation of distractor information because of capture is subsumed by the subsequent suppression, and thus that the suppression can be interpreted as a proxy index of capture. The current results challenge this account by showing that the reward-associate distractor is suppressed very quickly in the poststimulus interval, leaving little opportunity for prior selection. It is important to point out that, even within this new interpretation, evidence of suppression — here in the EEG signal, there in the fMRI signal — remains a valid index of the existence and strength of incentive salience. Naturalistic visual objects imbued with incentive salience are attention drawing and need to be strategically suppressed if they are not to be selected.

Incentive salience is thought to be of key importance to human addictive behavior (Robinson and Berridge, 2008). Direct drug stimulation of the midbrain dopamine system is thought to lead to the attribution of incentive salience to drug-related objects and environments. When these objects and environments are encountered in the future, they become difficult to ignore and, once noticed, induce craving and drug-seeking behavior. In line with this, many studies have reported that task-irrelevant, drug-related stimuli interfere with task-relevant behavior (for review, see Field and Cox, 2008). However, meta-analysis suggests that the relationship between drug craving and attentional bias is not strong (Field et al., 2009) and the clinical relevance of attentional bias in addictive behavior is the subject of continuing debate (e.g., Christiansen et al., 2015). This may reflect the mediating influence of strategic attentional control on drug-induced attentional bias. If attentional bias to drug-related stimuli can be reduced through strategic attentional control, drug-related stimuli may be suppressed rather than selected (Garavan and Hester, 2007). This idea is consistent with the broad notion that addictive behavior is closely linked to reduced activity in inhibitory prefrontal brain regions (Goldstein and Volkow, 2002). It is also consistent with recent results from studies of attentional bias in restrained eating. Although results in the eating literature vary, some studies show that task-irrelevant images of high-caloric foods interfere with strategic behavior more strongly in unrestrained eaters than in restrained eaters, suggesting that restrained eaters strategically suppress processing of the food stimuli (Veenstra et al., 2010; Forestell et al., 2012; Werthmann et al., 2016; but see Meule et al., 2012; Neimeijer et al., 2013; for review, see Werthmann et al., 2015). A core puzzle in our understanding of addiction and eating disorders is that the same experiences and context can lead to dire disorder in one individual, but leave another unscathed, and there is clear opportunity for research on the strategic attentional control of incentive salience in mediating these outcomes.

In conclusion, we demonstrate that prior reward association can cause examples of a category or real-world objects to become salient and attention-drawing. However, these objects do not necessarily capture attention. Participants can establish strategic attentional control over these stimuli, suppressing their representation without the preceding allocation of attention to their location. This neural mechanism for control over incentive salience appears to support adaptive, strategic information-gathering in the natural environment.
